# Status and prospects of genome-wide association studies in cotton

**DOI:** 10.3389/fpls.2022.1019347

**Published:** 2022-10-18

**Authors:** Muhammad Yasir, Hafiza Hamrah Kanwal, Quaid Hussain, Muhammad Waheed Riaz, Muhammad Sajjad, Junkang Rong, Yurong Jiang

**Affiliations:** ^1^ The Key Laboratory for Quality Improvement of Agricultural Products of Zhejiang Province, College of Advanced Agricultural Sciences, Zhejiang A&F University, Hangzhou, China; ^2^ School of Computer Science, Chongqing University of Posts and Telecommunications, Chongqing, China; ^3^ State Key Laboratory of Subtropical Silviculture, Zhejiang A&F University, Hangzhou, China

**Keywords:** GWAS, cotton, SNP, linkage disequilibrium, fiber

## Abstract

Over the last two decades, the use of high-density SNP arrays and DNA sequencing have allowed scientists to uncover the majority of the genotypic space for various crops, including cotton. Genome-wide association study (GWAS) links the dots between a phenotype and its underlying genetics across the genomes of populations. It was first developed and applied in the field of human disease genetics. Many areas of crop research have incorporated GWAS in plants and considerable literature has been published in the recent decade. Here we will provide a comprehensive review of GWAS studies in cotton crop, which includes case studies on biotic resistance, abiotic tolerance, fiber yield and quality traits, current status, prospects, bottlenecks of GWAS and finally, thought-provoking question. This review will serve as a catalog of GWAS in cotton and suggest new frontiers of the cotton crop to be studied with this important tool.

## Introduction

Cotton is one of the most important cash crops, accounting for approximately 35% of total fiber consumption worldwide. Most of the world’s cotton production comes from upland cotton (*Gossypium hirsutum*), which has a wide range of adaptability and high yield. Owing to a key component of the global textile industry, cotton fiber traits have gained more attention of researchers as compared to other traits. Cotton is grown in more than 30 countries as a major commercial commodity for food, feed, and renewable fiber (Ullah et al., 2017). It has evolved through a long process of polyploidization followed by diploidization. It is an ideal crop for studying a wide range of biological phenomena such as genome evolution, diversification, single-celled biological processes, biotic and abiotic stress tolerance (Qin and Zhu, 2011; Shan et al., 2014; Du et al., 2018).

Natural and human selection pressure generates natural variants of crops from their wild progenitors. The decoding of the cotton genome has provided valuable insight into the role of natural variations and polyploidy in the improvement of agronomic traits in the genus *Gossypium* (Zhang et al., 2008). About 5–10 million years ago, the ‘A’ diploid genome diverged from the eudicot progenitor along with the ‘D’ diploid genome (Wendel, 1989; Wendel and Albert, 1992). A transoceanic dispersal of an A-genome ancestor (*Gossypium arboretum* L.) to the New World crossed with a D-genome ancestor (*Gossypium raimondii* L.), resulting in allotetraploid cotton around 1–2 million years ago (Wendel, 1989). This strong evolutionary nexus between allotetraploid and diploid cotton genomes will assist us in better understanding the role of natural variations in association with yield, resilience to climate change, better adaptation, neofunctionalization and subfunctionalization of genes through the period of evolution of gene expression, as most of the wild, diploid and tetraploid cotton species have shared genetic functions (Wendel, 1989). Modern crop breeding necessitates a thorough understanding of the genetic basis of the origin, adaptation, and occurrence of natural genotypic and phenotypic variations to develop successful target-oriented breeding programs. The GWAS has enabled us to comprehend all of these multiple genetic patterns mediating complex traits.

**Figure 1:**
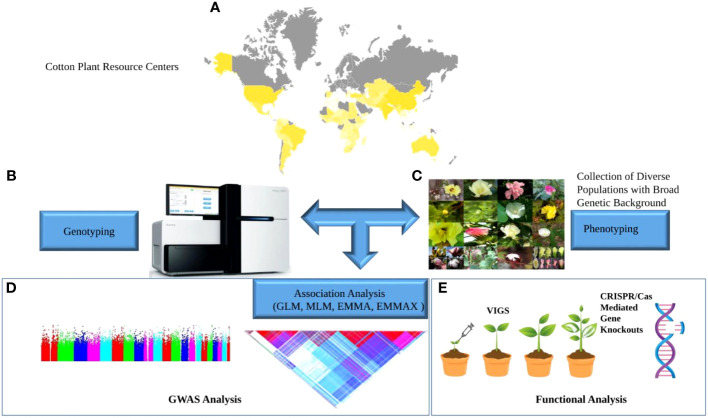
Schematic flow of GWAS. Collection of diverse germplasm; **(A)** Yellow shaded area on the world map depicts key cotton-growing countries; **(B)** Genotyping platform **(C)** Phenotypic diversity in cotton crop **(D)** GWAS analysis to find associated SNPs. **(E)** Functional validation of genes associated with traits under study.

Genome-wide association study (GWAS) is an experimental design to dissect the association of natural genetic variants and traits in samples representing a big population (Visscher et al., 2017). GWAS traces ancient genetic crossovers, allowing researchers to identify genetic loci underlying traits at a much higher resolution than previously possible. This technique got much attention and success in human genetics, coupled with advancements in sequencing technologies. It has become a powerful tool for identifying natural variations underlying the complex attributes of crop plants (Gupta et al., 2014). Compared to GWAS in humans, GWAS in crops have an advantage of a permanent population (natural population of diverse background or homozygous varieties) resource to be genotyped for once, that can be phenotyped for different traits (Atwell et al., 2010). A schematic flow of GWAS has been illustrated in **Figure 1**. **Figure 1A**, yellow shaded area of the map, shows major cotton-growing countries in the world; **Figure 1B** shows genotyping/sequencing platform, phenotypic diversity in cotton crop is sown in **Figure 1C**. GWAS, combined with advanced phenotyping systems has made it possible to investigate genetic diversity at the nucleotide scale precision.

Association mapping usually based on single nucleotide polymorphisms (SNPs) markers and phenotype of interest is used to find causative locus as shown in **Figure 1C**; DNA sequencing detects SNPs in different individuals/plants, comparing DNA sequences reveals common genome variations. Some DNA variations (haplotypes) are more common in certain traits. Haplotypes associated with a trait can pinpoint its gene(s). DNA sequences closer together on a chromosome tend to get inherited together and will often stay together over time. A haplotype is a set of close-together DNA variations (SNPs) on a chromosome that are inherited together. Because they’re so close, there aren’t many crossovers or recombination between these SNP groups. A haplotype can be a single gene’s alleles or multiple genes’ alleles. In-between-gene SNPs are possible too. Basically, it just means that these are variations in the DNA that are so close together that they tend not to recombine, and therefore tend to be passed down through the generations together. the colored triangle shows linkage disequilibrium LD block **Figure 1D**, small red triangles shows the presence of SNPs or group of SNPs having a strong association with other SNPs. Association analysis of the phenotype of interest and SNPs are shown in the Manhattan plot (to show association statistical significance –log10 *P*-value) **Figure 1D**. GWAS relies on linkage disequilibrium (LD) between markers and functional variations of causative genes. The probability of gametic-phase separation of two closely located loci by recombination is relatively less often than the loci located apart from each other. This nonrandom association or linkage of two loci is known as LD. SNPs near the causative loci can be in high association with the functional variation and thus associated with the phenotype under study. GWAS identify these associations and genomic regions containing these significant SNPs and the implicated genes. One of the interesting aspects of GWAS is the identification of pleiotropic genes (the genes controlling more than one trait at a time). There are several types of pleiotropy, however GWAS studies report pleiotropy regardless of the specific type. Identification of large effect pleiotropic regions can assist in modification the modification of multiple traits with less effort and resources. However, functional validation of associated genomic regions SNPs/genes is necessary for the fruitful utilization of GWAS results; for that purpose, virus-induced gene silencing VIGS or the most advanced and precise genome editing CRISPR/Cas technique can be used, **Figure 1E**.

A genome-wide association study’s success and robustness lies in four sound bases: Genetic diversity, trait acquisition veracity, marker density, and statistical methodologies. GWAS has now been successfully applied in several crops, and a considerable number of studies have also been conducted on the cotton crop (Rice et al., 2020; Li et al., 2021b; Li et al., 2021c; Somegowda et al., 2021; Zhang et al., 2021; Zhong et al., 2021). In this review, we aim to provide an overview of GWAS in cotton, the success of GWAS, shortcomings, future perspectives, and finally, thought-provoking questions.

## GWAS in cotton

**Figure 2:**
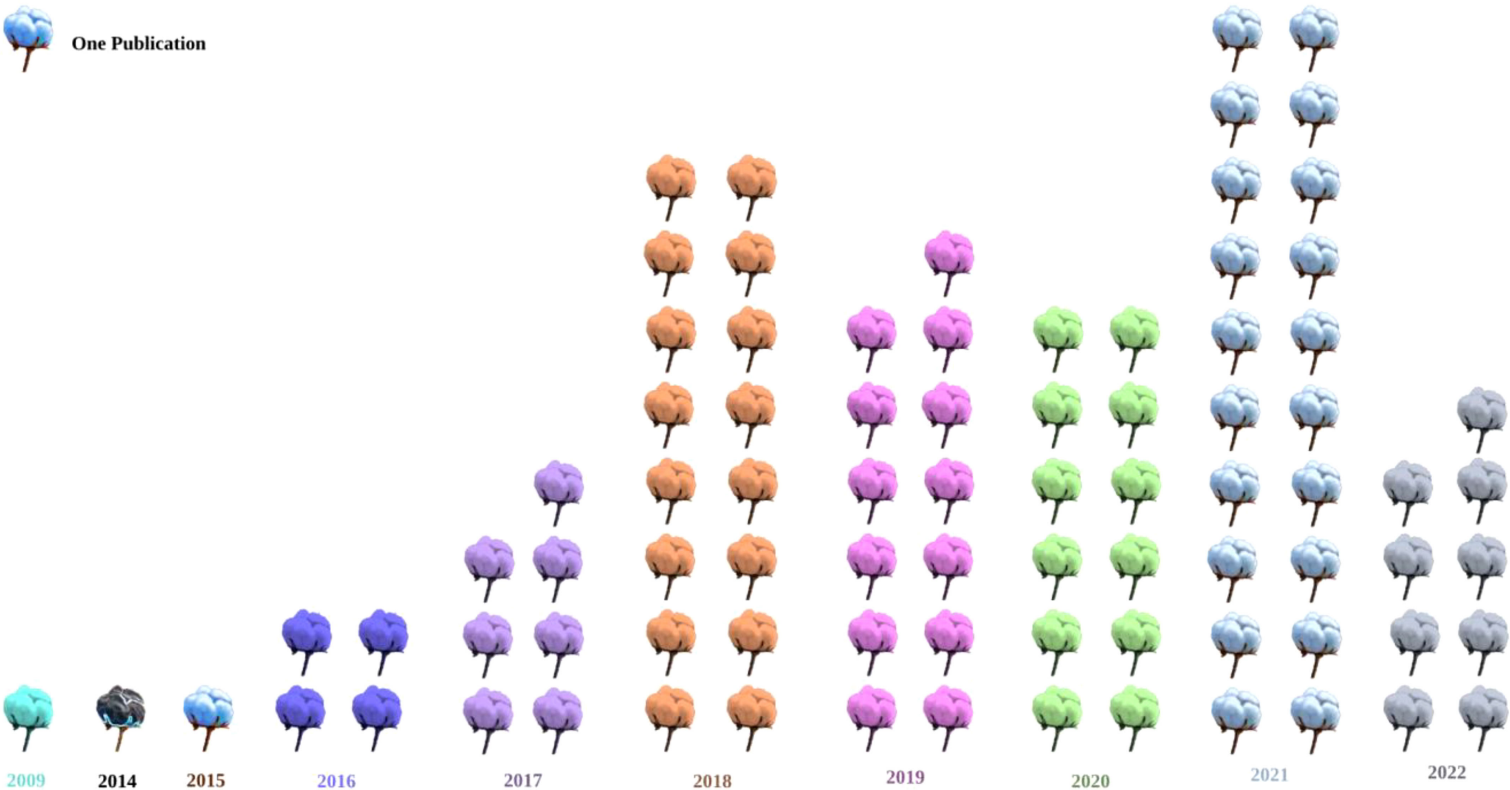
An increasing number of GWAS publications from 2009-22. Each opened boll represents one publication, specific color depicts publications of a specific year.

GWAS, also known as linkage disequilibrium (LD) mapping, uses the phenotypic and genotypic variations within a species to identify the genetic underpinnings of the traits of interest. **Figure 2** provides an overview of the year-wise number of GWAS publications on the cotton crop.

**Figure 3:**
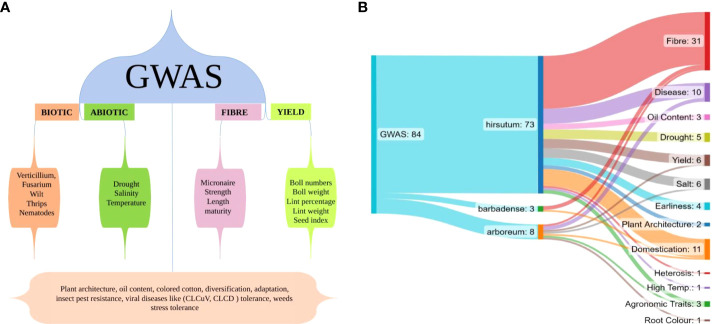
**(A)** This figure illustrates the GWAS conducted for different aspects of cotton, the bottom part shows less studied traits of cotton crop with GWAS tool; **(B)** This illustration shows total number of GWAS studies in all Gossypium species till now and node ends show total number of studies on a particular trait in different Gossypium species.

GWAS comes up with an opportunity to investigate the relationship between natural variations and major agronomic attributes of the cotton crop. Researchers have focused mainly on fiber, yield and abiotic stress in cotton crop, as shown in **Figure 3A**, however many other important aspects such as plant architecture, oil content, colored cotton, diversification, adaptation, insect pest resistance, viral disease tolerance and weed stress tolerance still need considerable attention, as shown in the lower portion of **Figure 3A**. Till now, 82 GWAS studies have been reported in cotton crop comprising 72 studies in *hirsutum*, 8 in *arboreum* and 2 in *barbadense.* GWAS studies for different traits in different species have been shown in **Figure 3B**. In the next section, we will discuss important GWAS studies conducted on different aspects of the cotton crop.

## Fiber quality traits (FQTs)

Cotton is an important industrial crop because of its natural spinnable fiber used in the textile industry. Diverse interspecific variations of fiber quality traits (FQTs) particularly fiber length trait (fuzless to extra-long fiber), depicts the genetic complexity of this trait. Low FQTs lowers the market value of cotton lint. Considerable research and breeding efforts have been expended on identifying and utilizing allelic variance that contribute to FQTs improvement. The development of cotton varieties with improved FQTs is highly desirable. More than 30 GWAS studies have been conducted on FQTs till now. Ma et al. (2018) resequenced a core collection of 419 genotypes of a natural population for 13 fiber traits in 12 diverse environments and evaluated for genomic variations with 3.96 million SNPs. 7383 SNPs depicted association with fiber traits, elucidating sufficient genomic variation present in the population under study. Single nucleotide polymorphisms (SNPs) associated with fiber traits were more prevalent than any other trait. Association of more number of SNPs with fiber traits than any other traits shows that genes involved in fiber traits improvement were under positive selection pressure. Consequently, positive selection pressure increased the proportion of fiber quality attributes. The identification of association of several important genes like *Gh_D03G0728* coding for COP1 interactive protein *CIP1* regulating the flowering time, *GhUCE* involved in fiber initiation, *GhFL2* a pleiotropic gene-regulating both fiber length and strength and some previously unreported genes associated with fiber length and other FQTs presents an excellent example of the power of GWAS. Most SNPs were found on the *D* subgenome. *COP1* in Arabidopsis is involved in mediating light-dependent growth and development of floral organs. The function of COP*1* (designated as *GhCIP1)* in *G.hirutum* was unknown, so GWAS was found useful tool to identify genes with previously unknown function. The results of Virus-induced gene silencing (VIGS) for functional validation of *Gh_D03G0728* containing different haplotype non-synonymous SNPs further corroborated the GWAS results. Silenced plants (plants with silenced gene under study) had not produced any flowering or squares; however, non-silenced (non-VIGS or CK) plants produced three fruiting branches having squares at the same growth stage, this functional analysis validated the exact effect of gene associated with flowering, suggesting it to be a key lead gene regulating flower development. A team of researchers conducted GWAS study and examined 196 cotton accessions with 41,815 SNPs. Two different GWAS models, a single-locus model [MLM (Q+K)] and five multi-locus models, were employed in six different environments for five fiber-related traits. Both the models commonly found 40 SNPs, 38 quantitative trait loci QTLs and 89 candidate genes for fiber traits. Moreover, 13 QTLs and 5 putative genes were reported with probable pleiotropic effects (Yuan et al., 2019). Single locus GWAS model and multi locus GWAS models have their own uses and limitations, in single locus model a key concern is the high false positive rate especially in large field experiments. To reduce false positive rate, Bonferroni correction is frequently applied in the single-locus methods which results in many important loci associated with the target traits being eliminated because they do not satisfy the stringent criterion of the significance test. However multi-locus models do not require Boneferroni correction thus more marker-trait associations are identified (Li et al., 2018a). Concurrent application of different GWAS models in cotton crop reveal the suitability of GWAS in cotton crop to decipher natural variants associated with complex traits.

Most of the studies have reported an inverse relation between FQTs and the final yield of cotton. However, an increase in lint percentage is directly proportional to cotton yield. *Gh_D05G0313* lycopene cyclase family gene, located on chromosome D05 coding for phytoene synthase (a key regulator enzyme in the biosynthesis of carotenoids), was found to be associated with lint percentage (Song et al., 2019). The same gene has been well characterized and reported to be involved in enhanced provitamin A production in cottonseed oil (Yao et al., 2018). Fine mapping of genomic region containing SNP associated with lint percentage and oil content can assist in breeding cotton varieties with enhanced oil content and lint percentage. Utilization of genomic regions containing pleiotropic genes can be a cost effective method to foster breeding programs. In addition, another gene, *Gh_D05G1124*, Probable protein phosphatase 2C 21, has been found to be associated with lint percentage. Moreover, GWAS deciphered 23 SNPs, and 15 QTLs with a strong lint percentage association in a panel of 276 cotton accessions genotyped with Cotton60k SNP array. Most of the loci were located on the *Dt* subgenome than *At* subgenome (Song et al., 2019). It can be inferred that GWAS can provide complementary evidence about the pleiotropic effect of genes interaction.

Two different studies conducted by Wang et al. (2021) and Su et al. (2019) scoured FQTs and lint percentage using restriction site-associated DNA sequencing (Rad-seq) and specific locus amplified fragment sequencing (SLAF-seq) in 316 and 160 accessions of cotton, respectively. As a result of GWAS findings, 231 SNPs were associated with Fiber and yield-related traits in 27 genomic regions. Interestingly four genomic regions held favorable pleiotropic loci and 6 genes, D11 chromosome was found to be having most of the loci related to FQTs. Su et al. (2019) applied one single locus and six multi-locus GWAS models in four different environments. It was found that 4 and 45 quantitative traits nucleotides (QTNs) were associated with lint percentage in single-locus model SLM-GWAS and multi-locus model MLM-GWAS, respectively. Half of the identified QTNs were located on the *D* genome. Although several SNPs have been identified using different GWAS models, however the dire need is to validate genomic regions/genes containing highly associated SNPs as we know that GWAS only reports associated SNPs not causal SNPs, so we further need to confirm associated SNPs to declare them as the causal SNPs.

Cotton fibers are single celled highly thickened epidermal cell extensions of cotton seeds that make it a suitable model to understand single cell processes. Hence, FQTs are the most important and interesting area of research in the cotton crop, as fiber traits depend on the primary cell wall differentiation followed by pure cellulose synthesis during secondary cell wall thickening/maturation. In this section we have seen that most of the significant SNPs and candidate QTLs related to FQTs were located on the *D* subgenome. Later we will discuss the possible reasons for this feature. In this section, it has also been observed that *D* subgenome have more SNPs associated with FQTs than *A* subgenome. This finding suggest that *D* subgenome holds the promise of providing more useful knowledge to understand genetic patterns mediating FQTs.

None of the studies described above have employed large scale germplasm > 1000 accessions for the exploration of genetic diversity to connect with breeding programs. It has been proposed that GenBank genomics can serve as a link between genetic diversity and breeding in agricultural crops. Recent studies in different crops such as rice, wheat and watermelon have utilized large scale germplasm >1000 accessions to find complete set of novel variation, role of introgression in shaping adaptability and candidate genes for important traits respectively (Wang et al., 2018; He et al., 2019; Zhao et al., 2019). To bridge the gap of Genbank genomics study in cotton He et al. (2021) explored 3248 tetraploid cotton accessions genomes to decipher the genomic basis of geographic differentiation and fiber improvement. The genomes of 2500 cultivars were compared with their most probable exotic donors to find introgression events. The most introgressed fragments were found in *G.barbadense* widely distributed on all the chromosomes. Chromosome A09 was enriched with introgressed fragments in case of *G.arboreum*. Diploid specie *G. thurberi* contained highest introgressed fragments on D08 chromosome. Two large effect pleiotropic alleles *FL3* and *FS2* associated with fiber length and fiber strength overlapped with 61.8-62.2 Mb introgressed region of A09 chromosome named GaIR_A09 in *G. arboreum.* The accessions carrying GaIR_A09 showed significantly improved fiber length and strength than those lacking this fragment. GaIR A09 may be unique candidate locus introgressed from *G. arboreum* responsible for fiber quality in contemporary cultivars.

As described above, GWAS conducted by Ma et al. (2018) has provided reliable information about genes mediating flowering and fruiting branches. Gene expression was not only confirmed with RNA expression data but also functionally validated with virus induced gene silencing method. The association information in this study provides an opportunity to select potential genotypes/cultivars with exact haplotype that can be inducted in breeding program for improved fiber quality cultivars. Moreover, these findings suggest hotspots for molecular selection and genetic manipulation in cotton fiber quality improvement breeding programs. The identification of pleiotropic genes with GWAS further diversifies its application in a broader spectrum.

Cotton breeding programs depend on predefined breeding objectives, and the textile industry needs fine fiber, whereas farmers’ desire is to get high-yielding varieties. However, most of the FQTs are negatively associated with fiber yield traits. Here we will see the genetic variations underpinning the yield attributes that will help design cotton breeding programs better.

## Fiber yield traits

Due to its superior yield and adaptability, *Gossypium hirsutum* L. outperforms other cultivated species of cotton in lint production and meets more than 95% of total fiber demand (Chen et al., 2007). Cotton breeders have long sought to contrive high-yielding cultivars. Cotton yield improvement is hampered by a narrow genetic background and conventional breeding practices (Zhang et al., 2008). Investigation and pyramiding the elite quantitative trait loci (QTLs)/genes related to yield components for molecular breeding to boost cotton output is crucial. Molecular markers and biparental linkage mapping analysis have found many QTLs for cotton yield-related traits (Shen et al., 2007; Liu et al., 2012). However, due to the restricted number of markers and the huge QTL regions, it isn’t easy to utilize QTLs with marker-assisted breeding. An unprecedented advancement in sequencing technologies and statistical techniques have made it possible to exploit genomic variations at single nucleotide level. GWAS have emerged as an important tool to utilize the genomic variations to explore the genetic underpinnings of cotton yield traits employing SNPs. In the previous decade, assembly, sequencing and fine mapping of the cotton genome have accelerated the pace of gene mapping for key traits of cotton improvement (Li et al., 2015; Liu et al., 2015; Zhang et al., 2015; Hu et al., 2019; Wang et al., 2019). GWAS has utilized reference genome to identify several SNPs and candidate genes related to cotton yield (Fang et al., 2017; Wang et al., 2017; Ma et al., 2018; Song et al., 2019).

Genotypic data comprising more than 56 thousand SNPs explored the genetic factors associated with yield- traits in a population of 242 cotton cultivars (Zhu et al., 2021). GWAS identified 560 QTNs associated with yield traits in multiple locations. In total, 95 stable QTLs (sQTLs) (spanning two or more environments) with 12,23,45 and 33 QTLs associated with boll numbers (BN), boll weight (BW), lint percentage (LP) and seed index (SI) were found respectively. Identification of several sQTLs across broad range of agrometeorological environments and multiple years are considered important for breeders to utilize in marker assisted selection. One of the proposed mechanisms of *TPR* in cotton fiber development is that it forms a complex with actins to control fiber growth. Zhu et al. (2021) studied 242 cotton accessions in 13 different locations and reported 92 high-quality QTLs associated with four fiber yield-related traits, including 12 sQTLs and an important gene, *Gh_A07G1389*, controlling short fiber development in the stable QTL19 region encoding a tetratricopeptide repeat (TPR)-like superfamily protein (Zhu et al., 2021). Understanding how fiber cell elongates using a fiber mutant has been a fascinating subject of investigation (Hinchliffe et al., 2011; Ding et al., 2014; Gilbert et al., 2014). A separate study conducted by Fang et al. (2020) found a mutation induced in tetratricopeptide repeat (TPR)-like protein gene causing the short fiber phenotype in cotton. So we can infer that the results of GWAS study were in compliance with the study based on functional validation of gene coding same protein as found in GWAS. Pentatricopeptide repeat gene *PPR* similar to *TPR* gene has been well characterized in cotton fiber development (Thyssen et al., 2016). The amino acid composition and structure of Pentatricopeptide proteins are similar to those of tetratricopeptide repeat proteins. Few studies have been reported on the role of *TPR* gene in fiber development, however GWAS have reported association of TPR gene with fiber development.

A separate study explored 719 accessions of cotton genotyped with an SNP63K array with 63,058 SNPs. 62 SNP loci located mostly on *D* subgenome were associated with different fiber traits. Furthermore, two pleiotropic genes *(Gh_D03G1064* and *Gh_D12G2354*) that increased lint yield were also identified (Sun et al., 2018b). The protein coding gene *Gh D03G1064* encodes the FRIGIDA protein; a developmental protein in terms of molecular function, whereas differentiation and flowering protein in terms of biological process, involved in the regulation of flowering time in the late-flowering phenotype. It contributes to the enrichment of a WDR5A-containing COMPASS-like complex at the ‘FLOWERING LOCUS C’ that trimethylates histone H3 ‘Lys-4,’ resulting in FLC up-regulation and increased RNA levels (Jiang et al., 2009). A GWAS has successfully elucidated the role of two ethylene-pathway-related genes linked to higher lint yield and have a pleiotropic relationship with two fiber characteristics, LP and NB at A02:79153947 and D08:3040023 association signals, respectively (*GhLYI-A02* and *GhLYI-D08*). One non-synonymous SNP significantly associated with a trait of interest located in the gene *Gh_A02G1392*, a homolog of the *AP2/ETHYLENE RESPONSE FACTOR (ERF)-*type transcription-factor-encoding gene *AINTEGUMENTA-like 6 (AIL6)* in Arabidopsis. Comparative RNA-seq and (qRT-PCR) data showed high expression of this gene during seed development from 20 to 25 days post-anthesis, more than tenfold higher in TM-1 (lower lint percentage and number of bolls per plant) than in the corresponding Chinese improved cultivar ZMS12, which has a higher lint percentage and increased number of bolls per plant, in developing ovules at 10 and 20 DPA. The orthologous gene *AIL6* has a major role in shoot and flower meristem maintenance, flower initiation, organ size and floral organ identity, and cell proliferation in Arabidopsis. It has been reported that *ethylene insensitive 3 like protein* (*EIL*) serves as a key downstream regulator, *EIN3*, in the ethylene-initiated signaling network and binds to the promoters of *ERF* genes such as *AIL6*, stimulating their transcription in an ethylene-dependent manner. When a plant responds to both biotic and abiotic challenges, ethylene is a key phytohormone for orchestrating the integration of environmental signals into a specific phenotype (Dubois et al., 2018). However, ethylene also participates in cotton fiber development (Shi et al., 2006; Li et al., 2007; Qin et al., 2007). Higher accumulation of 3 transcripts of ethylene biosynthesis gene at fiber elongation stage, enhanced fiber elongation by exogenous application of ethylene, elimination of fiber elongation inhibition caused by 2-chloro-N-[ethoxymethyl]-N-[2-ethyl-6-methyl-phenyl]-acetamide (Qin et al., 2007), with ethylene elaborates the key role of ethylene in cotton fiber development. These findings imply that the equivalent multiple-functionality alleles linked to the ethylene pathway were rigorously selected during breeding of higher lint percentage (Fang et al., 2017).

A recent GWAS of 103 cotton accessions (breeding lines, released and obsolete cultivars) across three seasons identified the associations of SNPs to a wide range of cotton yield components, including fiber quality, plant architecture, and stomatal conductance. Out of 63,058 markers, only 28,480 SNP markers > 5% MAF were used for GWAS. For fiber length and micronaire, MLM revealed 17 and 50 significant SNP associations, respectively (Gapare et al., 2017). The possible reasons for less number of associated SNPs suitable for GWAS analysis in this study could be less DNA polymorphism or low resolution provided by Chip-seq in cotton population under study, because the chip seq platform is limited to detect only those SNPs which are exclusively present on the chip. Few SNPs associated with multiple traits revealed that GWAS was actually unable to find rare variants, genetic interactions and genetic variance heterogeneity. GWAS detects common variants in a germplasm collection but has limited power to detect rare variants. Plant breeders are sometimes interested in finding rare variants. A possible solution for such germplasm populations with less genetic diversity is to use whole-genome selection (WGS) prediction instead of GWAS. WGS forecasts the performance of new quantitative trait candidates by combining hundreds or thousands of SNP markers with prior phenotypic data.

Another GWAS of 231 recombinant inbred lines RILs genotyped with 122 SSR and 4,729 SNP markers in nine locations was conducted for fiber and yield quality characteristics. The cottonSNP80K array platform, having 77,774 SNPs, was used for genotyping. There were 134 QTLs for fiber traits and 122 QTLs for yield parameters; 57 of these QTLs were sQTLs. Four multi-locus GWAS models identified a total of 209 and 139 (QTNs) linked with fiber yield components, respectively. A total of 51, 50, and 38 QTNs were found to be linked with BW, LP, and SI, respectively, with 82, 83, and 65 candidate genes. KEGG analysis revealed two yield-related candidate genes involved in six pathways. The putative genes found in 57 sQTLs were matched to those found in QTN, 35 common candidate genes were reported with four possibly pleiotropic genes (Liu et al., 2018). Yield can either be increased by improving complementary factors involved in lint production or by decreasing the effect of limiting factors like biotic and abiotic stresses. GWAS application in multiple seasons and locations have provided several clues about candidate SNPs and genes involved in yield improvement.

Identification of *TPR* gene associated with fiber yield traits using GWAS and functional validation of *TPR* transgene of Arabidopsis in *G. hirsutum* in a different study confirms the results of GWAS. So, this study supported by previous studies of molecular characterization of *TPR* gene, suggests the application of GWAS for the prioritization of candidate genes to develop transgenic plants with increased fiber yield. Previous studies have generally described the role of ethylene in abiotic stress tolerance; however Fang et al. (2017) deciphered the role of ethylene phytohormone in fiber elongation using the GWAS tool. This study promises to utilize a single gene involved in a cascade of biological pathways to develop abiotic stress tolerant genotypes with long fiber characteristics. The need of the hour is to do functional validation of the potential candidate genes identified with GWAS and field evaluation of the germplasm containing causal/associated SNPs/genes.

## Biotic and abiotic stresses

Plants have evolved intricate molecular pathways to cope with biotic and abiotic stresses. The dynamic climatic conditions have led to exceptional weather patterns resulting in augmented crop losses. Similarly, disease dissemination of pathogens in an era of increased international commerce has resulted in pathogen introduction and adaption to new places, resulting in recurrent outbreaks. A deep apprehension of the molecular processes underpinning plant stress responses to generate climate-smart crop varieties is becoming increasingly important (Prasad et al., 2021). Environmental stresses can be categorized into biotic and abiotic stresses. GWAS has provided a tremendous opportunity to explore the complex genetic patterns and genes involved in stress tolerance biological pathways. Here we will elucidate some studies that describe the application of GWAS to decipher the molecular mechanism of biotic and abiotic stress resilience in cotton crops.

*Verticillium wilt* is a plant disease caused by a soil-borne fungus, *Verticillium dahliae*. It is a severe disease in cotton (Sink and Grey, 1999; Klosterman et al., 2009). *V. dahliae* outbreaks in cotton have become more common in China, the disease is prevalent on nearly all cotton acreage, resulting in massive economic losses every year (Mo et al., 2016). To identify potential loci implicated in wilt resistance, GWAS in 215 Chinese *Gossypium arboreum* accessions inoculated at seedling stage with *V. dahliae* have traced 309 loci significantly associated with *Verticillium wilt* resistance with the strongest signal Ca3 in a 74 kb haplotype block. *CG05*, designated as *GaGSTF9*, was located close to the most significant SNP Ca3 23037225 (14 kb); this gene has been more responsive when treated with *verticillium dahilae* and salicylic acid (SA). Therefore, it was inferred that *CG05* might respond to invasion by *V. dahliae via* an SA-related signaling pathway. *GaGSTF9* was found to be a positive regulator of *Verticillium wilt* through the use of (VIGS) and overexpression in Arabidopsis. Genes identified in GWAS studies followed by functional validation provides robust and valid results in terms of molecular markers that can be utilized in the development of genetically modified plants with improved characteristics.

Moreover, the Arabidopsis mutant *gstf9* of (GST) was more susceptible to *Verticillium wilt* than wild-type plants. The endogenous SA and H_2_O_2_ expressed a significant effect on Arabidopsis that overexpressed *GaGSTF9*, depicting that GST may regulate reactive oxygen species (ROS) content *via* catalytic reduction of the (GSH), subsequently affecting SA content. Plant *glutathione S-transferase* (*GSTs*) genes have been classified into several classes, including dehydroascorbate reductase (DHAR), metaxin, phi, tau, zeta, theta, iota, lambda, and others. In cotton, few types of glutathione S-transferase GSTs have been identified. These findings elucidate the success of GWAS in identifying *GaGSTF9* gene, a key regulator mediating cotton responses to *V. dahliae* and a potential candidate gene for cotton genetic improvement (Gong et al., 2018).

*Verticillium wilt* foliar disease severity ratings (DSR) indexed, multi-parent advanced generation inter-cross (MAGIC) population of 550 recombinant inbred lines (RILs) coupled with 11 upland cotton parents with a total of 473,516 polymorphic SNP markers used to identify chromosomal regions for VW resistance, GWAS identified three QTLs on chromosome A01, D02 and D08 in common and three QTL clusters detected on chromosome A01, A13, and D01. Potential candidate genes for VW resistances were found in a narrow region of three common resistance QTLs (Zhang et al., 2020). Identification of common QTLs on different chromosomes link certain complex phenotypes to specific regions of chromosomes. Common QTL regions associated with particular trait of interest can be skewed towards fewer QTLs with major effects (Hayes and Goddard, 2001).

A combined study of GWAS, QTL-seq and transcriptomic analysis showed the role of the flavonoid biosynthesis pathway in VW resistance in cotton crop. Gene *Ghir_A01G022140* encodes a pyridoxal 5′-phosphate synthase subunit PDX1 involved in vitamin B6 biosynthesis, which is important for disease resistance in tomato and *Arabidopsis thaliana*  (Zhao et al., 2021b). A comparative study of Combined results of GWAS, QTL-seq and transcriptome sequencing detected basal defense-related genes showing gDNA sequence and expression variation in VW tolerant and sensitive cotton lines, which might be the molecular mechanisms of VW resistance in *G. hirsutum.* Vitamin B6 is an assembly of six vitamers: pyridoxine (PN), pyridoxal (PL), pyridoxamine (PM), and their phosphorylated derivatives. The *de novo* biosynthesis of VB6 vitamers requires two highly conserved pyridoxal protein families (PDX1 and PDX2) in plants. Vitamin B6 has a crucial role in stress responses as it is involved in pathways of starch metabolism, glucosinolate biosynthesis, ethylene and auxin biosynthesis (Mooney and Hellmann, 2010). In previous studies, plants with complete PDX knockouts have depicted increased sensitivity with B6 reduction to high levels of salt, sucrose, mannose, polyethylene glycol (PEG), UV and different diseases (Titiz et al., 2006). In contrast, transgenic plants with overexpression of PDX showed higher tolerance to different biotic and abiotic stresses, which corroborates the role of B6 in plant’s defense systems (Raschke et al., 2011). These findings depict that multilayered (GWAS, QTL, transcriptomic) identification of genes for traits under study provides more robust results.

*G.hir_A01G022110*, a negative-regulated gene coding for 2-oxoglutarate-dependent dioxygenase involved in the flavonoid biosynthesis pathway, has been linked to VW resistance by the enrichment of flavonoids in a spontaneous cotton mutant with red coloration, which results in a significant increase in resistance to *Verticillium dahliae*, these findings, supported by metabolomics studies, revealed the success of GWAS in figuring out the multifaceted genetic pattern of disease resistance mechanism in cotton crop.

Cotton is an evergreen perennial crop attracting numerous insect pests. Insect pests cause great damage by attacking leaves, bolls and consequently causing reduction in yield. Only a few GWAS studies have been done to find genetic association for insect resistance mechanisms in cotton crop. Evaluation of an association mapping panel of 376 Upland cotton accessions identified quantitative trait loci (QTL) for Thrips resistance in two replicated tests. Based on a GWAS using 26,301 polymorphic SNPs Eight QTL were identified for Thrips resistance on five chromosomes, with most SNPs on the *D* subgenome (A09, D01, D02, D03 and D11) (Abdelraheem et al., 2021a). Exclusive presence of several QTLs on D genome is an indication of persistence of genomic regions on D genome over the course of selection and evolution of cotton crop. These genomic regions can help us to trace out signatures of selection for Thrips resistance in cotton crop.

Bacterial blight (BB), caused by *Xanthomonas citri* pv. *malvacearum* (Xcm) is a destructive disease of cotton crop in many countries. Breeding BB-resistant cotton cultivars is the most effective strategy for controlling this disease. A current study of GWAS in 335 cotton accessions has been reported. GWAS, based on 26,301 polymorphic (SNP) markers, detected 11 QTLs associated with 79 SNPs, including three QTLs located on each of the three chromosomes, A01, A05 and D02, and one QTL on each of D08 and D10. Once again, these results found more associated SNPs on the *D* subgenome. Several studies concurrent findings of genomic regions associated with multiple traits on *D* genome offer some clues to focus on genomic hotspots associated with several traits. These results will assist in breeding cotton for BB resistance and facilitate further genomic studies in fine-mapping resistance genes to enhance the understanding of the genetic basis of BB resistance in cotton (Elassbli et al., 2021).

Previous studies of abiotic stress have not dealt simultaneously with the genetics and genomics of drought and salt tolerance (DT, ST). A MAGIC population of 550 RILs and their 11 Upland cotton parents used 473,516 SNPs to pinpoint QTLs for (DT) and (ST) at the seedling stage. Transgressive segregation in the MAGIC-RILs indicated tolerant and sensitive alleles recombination during the crossing-over process for the population development. A total of 20 QTL were detected for DT, including 13 and 7 QTL related to plant height (PH) and dry shoot weight (DSW), respectively, whereas 23 QTL were detected for ST, including 12 and 11 QTL for PH and DSW, respectively. Four QTLs were reported on chromosome A13, three QTLs on A01 for DT, four QTLs on D08 and three QTLs on A11 for ST. Nine QTL were shared by DT and ST, showing possible implication that the two stresses have a common genetic base. Salt and drought stress-responsive 53 candidate genes were identified. However, these findings need further validation with functional analysis of putative candidate genes. The QTL discovered for both DT and ST have significantly increased the number of QTL for abiotic stress tolerance that can be used for marker-assisted selection (MAS) to develop DT, ST cultivars and further genomic studies to identify drought and salt responsive genes in cotton (Abdelraheem et al., 2021b). Common SNPs identified with GWAS for drought and salt stress tolerance provide time and cost effective opportunity to focus on common QTLs for breeding drought and salt-tolerant cultivars simultaneously.

319 upland cotton accessions genotyped by more than 55 thousand (SNPs) for nine traits related to drought tolerance, using two datasets to identify QTNs with multi-locus random-SNP-effect MLM (mrMLM) reported a total of 20 QTNs distributed on 16 chromosomes. A compendium of 205 genes was induced after drought stress, combined results of (GWAS), RNA-seq and qRT-PCR verification, proposed four genes to be potential candidate genes for drought tolerance, *RD2* encoding response to desiccation 2 protein, *HAT22* encoding a homeobox-leucine zipper protein, *PIP2* encoding a plasma membrane intrinsic protein 2, and *PP2C* encoding a protein phosphatase 2C. *PP2C* is an important regulator of stress signal transduction pathway and is an excellent candidate gene to decipher complex stress tolerance traits (Schweighofer et al., 2004; Hussain et al., 2021). Silencing of *GhPIP2;7* gene in *Gossypium hirsutum* has shown decreased chlorophyll content, superoxide dismutase (SOD) and peroxidase activity, it depicts positive regulatory role of *PIP2;7* in cotton under salt stress (Guo et al., 2022). Zheng et al. (2021a) reported cloning of *PIP2* gene from *Canavalia rosea* and overexpression in *A.thaliana* increased the recovery and survival rate under elevated drought and salt stresses. The most surprising aspect of the study was *PIP2* mode of action in drought and salt stress tolerance by mediating water homeostasis instead of reactive oxygen species (ROS) transporter. This shows that GWAS findings are in compliance with functional studies of the same genes in model plants and cotton plants. These results will contribute to a better knowledge of the genetic basis of drought stress tolerance in cotton and prospective markers for breeding drought-tolerant cotton cultivars (Hou et al., 2018).

Previous studies have not utilized advanced image-based automatic phenotyping platforms to bridge the gap between phenotypes and genotypes with the GWAS tool. A team of researchers has recently utilized an auto-phenotyping platform to examine 119 digital traits to decipher genes regulating drought stress tolerance in a population of 200 upland cotton accessions at the seedling stage. The phenomics data identified 390 genetic loci by GWAS using 56 morphological and 63 texture i-traits. Some digital traits identified drought-responsive genes, including *GhRD2, GhNAC4, GhHAT22* and *GhDREB2*. Moreover, potential candidate genes *Gh_A04G0377* and *Gh*_ *A04G0378* were negative regulators of cotton drought response. Comparative analysis of phenomics, GWAS and transcriptomic data provides an exceptional resource to characterize key genetic loci with an unprecedented resolution that can predict future genome-based breeding for improved drought tolerance in cotton (Li et al., 2020). It is of great interest that two different GWAS studies on same traits found *RD2* and *HAT22* as common genes involved in drought stress tolerance as mentioned above. Together these results provide important insights into drought stress tolerance mechanism. Advanced phenotyping platforms utilizing artificial intelligence-based image analysis are potential future resources for precise and accurate phenotyping. Although phenotyping is as important as genotyping yet, most of the time, researchers give more importance to genotyping accuracy. This study is a classic example of utilizing cutting-edge technologies for both genotyping and phenotyping coupled with GWAS.

A population of 316 upland cotton accessions studied with GWAS using GLM and a factored spectrally transformed LMM. A total of eight, three, and six SNPs were linked to the euphylla withering score (EWS), cotyledon wilting score (CWS), and leaf temperature (LT), respectively. Interestingly, combined results of GWAS and RNA seq depicted that DEGs *WRKY70, GhCIPK6, SnRK2.6*, and *NET1A* induced by drought stress were found in the candidate region, regulating ABA, the mitogen-activated protein kinase (MAPK) signaling and the calcium transduction pathways (Li et al., 2019). RNA seq analysis further corroborated these findings by elucidating differential expression of these genes under normal and drought conditions; moreover, the expression of these genes was found to be induced by the drought stress.

Soil salinity is a serious threat affecting more than 800 million hectares of arable land worldwide. Plant growth and development is significantly hindered under salt stress. GWAS has been successfully utilized to identify SNPs/genes associated with salt stress tolerance mechanisms. A GWAS in 217 cotton accessions for salt tolerance related (ST) traits at the seedling stage performed on 2 years of phenotypic data and more than 50 thousand SNPs identified 27 SNPs scattered over 12 chromosomes associated with three ST traits. Among these, the associations on chromosomes A13 and D08 for relative PH, A07 for relative shoot fresh matter weight (RSFW), A08 and A13 for relative shoot dry matter weight (RSDW) were stable SNPs, indicating that they were likely to be sQTLs. A total of 12 salt-induced candidate genes were identified by GWAS and transcriptome analysis (Xu et al., 2021).

A genome-wide association conducted in a core collection of 419 accessions of cotton representing a vast genetic background, subjected to GWAS for various agronomic traits. Phenotypic variations depicted significant variation between salt-sensitive and tolerant accessions. 17264 significantly associated SNPs were found distributed on multiple chromosomes. Twenty potential candidate genes discovered around SNPs A10_95330133 and D10_61258588, linked to relative water content under 150mM NaCl salt stress (RWC_150) and leaf fresh weight (FW_150). Fine mapping of these important genomic region can unveil candidate genes for functional validation. Differential expression of candidate genes under normal and drought stress conditions revealed the involvement of the genes in the salinity tolerance mechanism. Further study on the functional validation of candidate genes development of transgenic lines can provide useful knowledge about the genetic control of salt tolerance at the seedling stage (Yasir et al., 2019).

Zhu et al. (2020) examined three major components of lint yield across 316 *G. hirsutum* accessions over two years under four salt conditions. GWAS analysis reported 57,413 SNPs above *P*-value threshold. A total of 42, 91 and 25 sQTLs were associated with single boll weight SBW, lint percentage LP, and number of bolls per plant NBPP, respectively. Eight sQTLs discovered concurrently in four different salt environments in the case of LP, whereas SBW and BNPP had fewer sQTLs. According to gene ontology (GO) analysis, their regulatory mechanisms were also quite different. The transcriptomic analysis defined 8 genes associated with LP under salt stress; Haplotype analysis showed an MYB gene *GhMYB103* with two SNP variants in cis-regulatory and coding areas substantially linked with LP. Moreover, 40 candidate genes from NBPP QTLs were salt stress-responsive (Zhu et al., 2020).

Here we describe another GWAS study in which a high-throughput CottonSNP80K array was used to identify genotyping in various cotton accessions. 77,774 SNP loci were synthesized on the array. In 288 *G. hirsutum* accessions for GWAS, more than 54 thousand SNPs (MAFs >0.05) related to 10 salt stress attributes were found, with eight significant SNPs connected with three salt stress variables. Two loci on D5 were significantly associated with chlorophyll content, one on A2 and four on D9 were significantly associated with melondialdehyde content, and one on A12 significantly associated with germination rate (Cai et al., 2017). Similarly, in another study, two ST characteristics were assessed in a population of 713 upland cotton seedlings. Infinium CottonSNP63K array discovered marker-trait relationships under salt stress. Across seven chromosomes, A01, A10, D02, D08, D09, D10, and D11, 23 SNPs were linked with a relative survival rate of seedlings and salt tolerance level. The *D* subgenome had most of the candidate genes. The D09 SNPs i46598Gh and i47388Gh were linked to both traits. 280 potential genes showed differential expression under salt stress. Many of these genes have been implicated in salt tolerance in plants *via* transcription factors, transporters, and enzymes. The differential expression of six candidate genes in salt-tolerant and sensitive cotton cultivars confirmed their role in salt tolerance (Sun et al., 2018a). This study elucidated that most of the SNPs were skewed on the *D* subgenome. These findings are important for improving our understanding of the complex salt tolerance mechanisms in *G. hirsutum*.

Many GWAS studies have been reported for abiotic stress tolerance in cotton crop at seedling stage, however number of studies under field conditions are limited. Dynamic gene expression is highly influenced by environmental variables. It is imperative to study GWAS results at field level to believe on the stable expression of genes under changing environmental conditions. However, phenotyping of crops under certain stress is challenging under field conditions because of inhomogeneity of certain stress, difficult differentiation of plant response against concurrent effect of different stresses. We have learned from the above descriptions that GWAS has been successfully conducted on different biotic and abiotic stresses. These findings provide valuable information about the genetic underpinnings of the stress tolerance mechanism and prove the robustness of the GWAS approach. However, in some studies, we have seen that GWAS could not find the unprecedented number of associated SNPs with traits under study. The possible reasons for this inability could be the less natural genetic variation enrichment of the population or the low resolution of the sequencing platform, e.g. Chip_seq. If the population under study doesn’t have enough natural variation common in different lines/landraces/RILs or varieties but has rare causal genetic variants, then GWAS shouldn’t be preferred over WGS as WGS have more power to find out natural genetic variants than GWAS. This knowledge suggest that we should carefully select populations to perform GWAS analysis. One of the suggestions to overcome this shortcoming is to use core collections of populations to make a representative population from a big germplasm. The genes identified in GWAS studies are very much important in terms of their annotation and pathways involved in stress tolerance mechanism. Genetic characterization, transformation and functional validation of these genes in model plants and confirmation in cotton plants can provide promising genes that can be utilized for the breeding programs of stress-tolerant cultivars.

## Domestication and traits improvement

*Gossypium hirsutum* L. adapted during polyploidization to generate a larger fiber production and endure harsh environments better than *Gossypium barbadense* L., which yields superior-quality fibers. The genetic and molecular underpinnings of these interspecies divergences were unknown globally. However, GWAS has opened new avenues to trace genetic footprints that lead to speciation, interspecific divergence, spatio temporal adaptation and the development of modern cotton cultivars.

Here we discuss the findings of Hu et al. (2019) about the diversification of *G. hirsutum* and *G. barbadense* species. Whole-genome comparative analyses revealed that driving forces of speciation and the evolutionary history of these species were species-specific alterations in structural variations, gene expression, and gene family expansion. These findings aid in understanding cotton genome evolution and domestication history. The genetics behind local adaptation and domestication can be found in interspecific genomic diversity. A recent study addressed the role of interspecific haplotypes and introgression in improving the agronomic traits of the cotton crop. Two allotetraploid Gossypium species (*Gb*) and (*Gh*) were cultivated independently. A combined result of three GWAS panels (one panel of 229 *Gb* accessions and two panels of 491 *Gh* panels) revealed that most functional haplotypes related to agronomic traits were highly divergent, depicting strong divergent improvement between *Gb* and *Gh* accessions. According to the interspecific haplotype map, six interspecific introgressions from *Gh* to *Gb* were strongly related to *Gb* phenotypic performance, accounting for 5%–40% of phenotypic diversity in yield and fiber quality. In addition, three introgressions in *Gb* overlapped with six linked loci, indicating that these introgression sites were under selection and stabilization during the course of improvement. A single interspecific introgression might increase production while lowering fiber quality, or vice versa, making it challenging to raise yield and fiber quality simultaneously (Fang et al., 2021). A similar study found 315 introgression events from *G.hirsutum* to *G.barbadense* causing population divergence and agronomic trait variations. Moreover pleiotropic gene controlling traits was found (Wang et al., 2022).

Cotton’s development and domestication are fascinating from economic and evolutionary perspectives. An intraspecific QTL mapping population of 466 F_2_ individuals from an intraspecific cross between the wild *Gossypium hirsutum *var. *yucatanense *(TX2094) and the elite cultivar *G. hirsutum* cv. Acala Maxxa, in two environments targeting domesticated cotton phenotypes, found only 22 stable QTLs (sQTLs) associated with phenotypic changes during domestication. Even though around half of the QTL were found in the *A*-subgenome, numerous critical fiber QTL were found in the *D*-subgenome, inherited from a species with unspinnable fiber. Many QTLs were environment-specific, with few shared across the two environments, demonstrating that QTLs related to *G. hirsutum* domestication were genomically clustered yet environmentally mutable. It was concluded that the evolutionary dynamics shaping sympatric speciation divergence and domestication in cotton are complicated and that phenotypic alteration was likely influenced by several interacting and environmentally sensitive variables (Grover et al., 2020; Li et al., 2021a).

Natural and artificial selection pressure in crop plants leads to modifications in genotypes and phenotypes for better adaptation to changing conditions. A chromosomal inversion phenomenon is supposed to be an efficient source of accumulation of favorable alleles to fine-tune population genetic architecture. Many studies have reported this phenomenon in population adaptation in dynamic environmental conditions (Jones et al., 2012; Lamichhaney et al., 2016 ;Sinclair-Waters et al., 2018). Recently a GWAS study to identify adaptive loci involved in cotton crop subgroup differentiation concluded similar results. Loci associated with environment adaptation had locus on divergent chromosomal regions of A06 and A08. Collinearity analysis of several assembled genomes of cotton proved the evidence of chromosome inversion. Inverted sequences on chromosomes suppressed homologous recombination, allowing desirable alleles to persist in the subsequent populations. This study revealed the cause of population divergence and the consequences of variation in its environmental adaptability. These findings shed light on the genetic basis of environmental adaptability in Upland cotton, potentially speeding up the designing of molecular markers for climate change adaptation in future cotton breeding programs (Dai et al., 2020).

A variation map for 352 wild and domesticated cotton accessions scanned 93 domestication sweeps encompassing 74 Mb and 104 Mb of the *A D* subgenomes, respectively; moreover, GWAS found 19 potential loci for fiber-quality characteristics. Asymmetric subgenome domestication was reported as the responsible agent for long fiber directional selection. Global investigations of DNase I–hypersensitive sites and 3D genome architecture, which relate functional variations to gene transcription, showed the consequences of domestication on cis-regulatory divergence. This study provides new insights into the evolution of gene organization, regulation and adaptation in cotton crop and should serve as a rich resource for genome-based cotton improvement (Wang et al., 2017). However, directional selection results in the loss of desirable variations for important traits of interest.

An important study conducted by Nazir et al. (2021) re-evaluated a landrace of *Gossypium hirsutum*, formerly known as *Gossypium purpurascens* to understand the genomic structure, variation, and breeding potential, providing potential insights into the biogeographic history and genomic variations likely associated with domestication. Paucity of large number of cultivars/accessions used in all the GWAS studies on domestication and adaptability makes the results less representatives of the entire story. However, He et al. (2021) resequenced and mapped the data of 3278 cotton accessions to the reference genome. Based on phylogenetic and principal component analysis classification of more than 3K accessions into eight subgroups shows the enrichment of variation lead by natural and artificial selection. Fixation statistics (*F_st_*) is the proportion of the total genetic variance contained in a subpopulation (the S subscript) relative to the total genetic variance (the T subscript). Low *F_ST_* values within improved subgroups (G3–G6) demonstrated that the genetic divergence was low (0.019–0.067) within improved cultivars, However, the average pairwise *F_ST_* values were much higher (0.425– 0.552) between improved cultivars and landraces when using G1 (landraces group) as comparison pair than other. Higher *Fst* values between improved cultivars and landraces depicts noticeable genetic differentiation. Maximum divergence and major haplotypes were traced on chromosome A06 and A08 of *G.hirsutum*. *De novo* assembled genome carrying haplotypes of interests showed large scale chromosomal inversions causing haplotype polymorphism. Divergence on chromosomes A06 and A08 is the most notable genomic signature in cultivated G. *hirsutum*. Population differentiation in animals and plants has been linked to large chromosomal inversions. This evolutionary mechanism allows species to adapt to new environments by repressing recombination to sustain favorable genotypes. These findings support the theory of chromosomal inversion-population differentiation in crops and define the haplotypes associated with geographic differentiation in cotton cultivars. Here we enlist GWAS studies conducted on cotton crop (**Table 1**) for different traits, identified SNPs and QTLs.

**Table 1:** A summary of GWAS aimed at identifying SNPs/QTLs/Genes or quantitative trait nucleotides QTN, that contribute to cotton improvement.

Cotton species	Population Size (accessions)	Years/location/environment	Traits	No. of unique SNPS QTLs/sQTLs	Chromosomal location	Reference
*G.hirsutum* L	419	6 locations 2 years	13 fiber traits	7,383	A10, A07, A08, and D11	(Ma et al., 2018)
*G.hirsutum* L	196	6 environments	5 fiber traits	23 QTLs	A2, A6, A7, A9, A10, A13, D1, D5, D6, D7, D8, D10, D11, and D12	(Yuan et al., 2019)
*G.hirsutum* L	276	6 locations 2 years	1 fiber trait	23 SNPs and 15 QTLs	D05	(Song et al., 2019)
*G.hirsutum* L	316	9 environments	8 fiber traits	231 loci	A06, A07 and D11	(Wang et al., 2021)
*G.hirsutum* L	242	13 environments	4 yield traits	95 QTLs	A08	(Zhu et al., 2021)
*G.hirsutum* L	719	8 environments	5 fiber quality traits	62 QTLs	Dt11 and At07	(Sun et al., 2017)
*G.hirsutum* L	231 RILs	9 environments	Fiber and yield traits	32 and 25 sQTLS		(Liu et al., 2018)
*G.arboreum* L	215	Green house	*Verticilium dahiliae*	309 SNPs	A03	(Gong et al., 2018)
*G.hirsutum* L	550	Green house	*Verticillium dahliae*	3 sQTLs, 3 QTLs	A01, D02, D08, A13, D01	(Zhang et al., 2020)
*G.hirsutum* L	376	Green house	Thrips	8 QTLs	A09, D01, D02, D03 and D11	(Abdelraheem et al., 2021a)
*G.hirsutum* L	335	Green house	Bacterial blight	7 QTLs and 4 sQTLs	A01, A05, D02, D08 and D10	(Elassbli et al., 2021)
*G.hirsutum* L	550RILs	Green house	Drought, salt	20, 23 QTLs	A13, A01, D08	(Abdelraheem et al., 2021b)
*G.hirsutum* L	319	Green house	Drought	20 QTNs		(Hou et al., 2018)
*G.hirsutum* L	200	Green house	Drought	390 QTLs, 71 sQTLs	A04, D11, D13	(Li et al., 2020)
*G.hirsutum* L	316	Green house	Drought	7 QTLs	A01, A05, A11, D03	(Li et al., 2019)
*G.arboreum* L.	215	Green house	Salt	143 SNPs	A02, A07,A09, A11	(Dilnur et al., 2019)
*G.hirsutum* L	217	Green house	SALT	27 SNPs, 2 sQTLs	A07,A08,A13, D08	(Xu et al., 2021)
*G.hirsutum* L	419	Green house	SALT		A10, D10	(Yasir et al., 2019)
*G.hirsutum* L	316	4 environment 2 years	Salt	158 sQTLs	A05, A08, A11, A12, D06, D07	(Zhu et al., 2020)
*G.hirsutum* L	713	Greenhouse	Salt	8 SNPs	A01, A10, D02, D08, D09, D10, and D11,	(Sun et al., 2018a)
*G.hirsutum* L	149	Greenhouse	Salt	27 SNPs	A01, D01, D08	(Zheng et al., 2021b)
*G.hirsutum* L,*G.barbadense*	229 and 491	3 environment 3 years	Domestication	111 and 119 Loci	A03, A05,A08, D05, D12	(Fang et al., 2021)
*Yucatenese* x Acala Maxa	466	2 environments	Domestication	120 QTLs	Multiple chromosomes	(Grover et al., 2020)
*G.hirsutum* L	419	3 environments	Domestication	45 SNPs	A06, A08	(Dai et al., 2020)
*G.hirsutum* L	352	3 environments	Domestication	19 loci	A12, D04	(Wang et al., 2017)
*G.hirsutum* L	288	12 environments 2 years	Heterosis	271 QTNs	D09	(Sarfraz et al., 2021)
*G.hirsutum* L	550	12 environments 4 location 4 years	Fibre traits	25 sQTLs, 14 hQTLs	A07, D11	(Wang et al., 2022)
*G.arboreum* L.	215	3 environment	Fiber traits	177 SNPs	A05, A14	(Iqbal et al., 2021)
*G.hirsutum*	185	2 environment 2 years	Senescence	63 QTNs	A02, D03, D13	(Liu et al., 2022)
*G.barbadense* L.	336	6 years 4 locations	Fiber and fusarium wilt	6241 SNPs	D03, A05, D11	(Zhao et al., 2021a)
*G.arboreum* L.	215	Greenhouse	Biomass	83 SNPs	A7, A11	(Hu et al., 2022)
*G.hirsutum* L.	196	7 environments	Fiber	84 SNPs	A10	(Xing et al., 2019)
*G.arboreum* L.	246	Greenhouse	Nematode	15 SNPs	A01, A02, A03, A05, A06, A07, A09, A12	(Li et al., 2018b)
*G.hirsutum* L.	169	2 locations 2 years	Early maturation	29 SNPs	A6, A7, A8, D01, D02, D09	(Li et al., 2018a)
*G.arboreum* L.	215	Greenhouse	Root color	225 SNPs	A02, A04, A08, A09, A13	(Zhao et al., 2021b)
*G.hirsutum* L.	196	2 years field location	Oil content	47 SNPs, 28 QTLs	D12	(Yuan et al., 2018)

## Pervasive pleiotropy

Several GWAS studies described above have found some common SNPs/genes associated with more than one trait called pleiotropic SNPs/genes. A single gene controlling more than one apparently unrelated distinct traits/phenotypes is called pleiotropic gene. Several studies have revealed pleiotropic genes regulating multiple variables in cotton crop, so we will outline the likely causes and advantages of pleiotropic SNPs/genes. One possible explanation for pleiotropy is that the product of a single gene can be utilized in different cell types or can participate in cascade-like signaling to different targets. It’s difficult to discern actual biological pleiotropy from mediated and pseudo pleiotropy because genes normally work in intricate pathways/networks causing associated phenotypes. Mutual pathway sharing of two associated phenotypes leads to mediated pleiotropy, while spurious pleiotropy occurs when identified SNPs lies in a small region of high LD, where two tightly packed distinct genes lie, that regulate two different variables/phenotypes. However, no GWAS studies have reported such a deep level of understanding of pleiotropic genes. Pleiotropic genes can be considered hot spots for precise genome editing with the most advanced techniques like CRISPR/Cas system to explore the potential effects on associated phenotypes. Cotton genome editing at loci having pleiotropic genes can provide an opportunity to fine-tune genomic regions controlling more than one trait with little effort. Collective regulation of multiple metabolic processes can be modified in single gene editing event instead of editing several genomic regions. Moreover, editing of pleiotropic genes can produce large effect novel phenotypes. We have discussed several GWAS studies reporting pleiotropic genes based on their action on seemingly unrelated phenotypes. Still, there is not a single in-depth study stating the validation of pleiotropic genes identified with the GWAS tool. As the number of GWAS reports of pleiotropic genes increases over time, cotton breeders will pay greater attention to it. There are no reports on the functional characterization of pleiotropic genes or SNPs found with the help of GWAS. It’s possible that with the development of new statistical models and genomic techniques for GWAS investigations, we will be able to learn more about pleiotropic loci.

## Genomic basis of fiber development and occurrence of more SNPs on *D* subgenome

It has been anticipated that the spinnable fibers were formed only once in the progenitor of the *A* genome. Although this long morphology evolved in the *A*-genome progenitor, it has been found that the *Dt* genome was also important in tetraploid cotton fiber formation. Even though the *D* genome ancestor does not produce spinnable fiber, nonreciprocal DNA exchanges from *At* to *Dt* have resulted in the recruitment of *Dt* subgenome genes into the regulatory processes of tetraploid cotton fiber development. *Dt* has 1.4 times (104 Mb) as many sequences containing domestication signals as *At*, demonstrating a case of asymmetric domestication for the two coexisting subgenomes. The *Dt* subgenome also has 2.2 times more regions with selection sweep signatures than the *At* subgenome, indicating that it has been subjected to more selection pressure than the *At* subgenome. As demonstrated by the large number of fiber quality-related QTL hotspots discovered in the *Dt* subgenome. Similarly, findings have reported that there were more fiber-related genes in the *Dt* subgenome than in the *At* subgenome (Xu et al., 2015). It stands to reason that a collaboration between the two coexisting subgenomes has improved the fiber quality and yield of current tetraploid cotton cultivars.

As it is evident from our previous description of SNPs that, most of the candidate SNPs or loci associated with different yield and FQTs have been found on the *D* subgenome. The probable reason for these findings may be that the D genome provides many fiber genes after merging with another parental diploid cotton (*Gossypium arboreum*) *A* genome during evolution and domestication, even though the *D* genome does not produce any spinnable fiber.

## Benefits and limitations of GWAS

GWAS have revolutionized the field of intricate genetic architectures of important agronomic traits over the past decade, providing numerous compelling associations for complex quantitative traits. Despite numerous successes in identifying novel genes and biological pathways and in translating these findings into breeding programs in different crops, GWAS has not been without controversy. Here we provide an overview of the benefits and limitations of GWAS (**Table 2**).

**Table 2:** A comparison of GWAS benefits and limitations.

BENEFITS	LIMITATIONS
No need to have prior knowledge of biological pathways of the traits under study. Possibility of discovering novel candidate genes that have not been identified using previous methods. Encourages the formation of collaborative consortia to recruit sufficient numbers of participants for analysis, which tend to continue their collaboration for subsequent analyses. Rules out specific genetic associations. Provides data on the ancestry of each subject, which assists in matching case subjects with control subjects. Data on two types of structural variants, sequence and copy number variations, is provided, resulting in more robust data. Identify genetic contributors to common, complex traits for which each gene may only have a small effect.	The findings must be replicated in independent samples from diverse populations. It is necessary to have a large study population. GWAS studies look for correlation rather than causation. GWAS pinpoints a specific site rather than entire genes. Many of the variations (SNPs) found in GWAS aren’t near a protein-coding gene The combined impact of many SNPs typically only explain a modest fraction for any given characteristic. Finding related variations doesn’t always reveal the trait’s underlying biology.

## Meta-GWAS a way forward in genome-wide association studies

Genome-wide association study estimates the statistical association of single nucleotide variants with variables of interest. Although the number of GWASs has exponentially increased in the previous decade, the results have low reproducibility. Different GWASs on the same trait are not in compliance with each other, which means different GWAS results report different genomic regions for the same trait. That might be because of insufficient genetic variation in mapping populations, and different algorithmic and statistical approaches. The potential reason behind this conundrum could be the following factors.

- Low accuracy of phenotyping
- Phenotyping of non/low heritable traits
- Inaccurate sample size in terms of variation
- Wrong statistical analysis
- Population stratification
- Technical biasedness

Meta-GWAS is a statistical technique to combine the results across the GWA studies. It has garnered considerable attention from academics to resolve disparities in genome-wide associations. Meta-GWAS has been widely employed in human studies with a large amount of available data. Such work is becoming more readily available for commonly used populations, such as haplotype and regional mapping populations. Online tools such as MetaGenyo (http://bioinfo.genyo.es/metagenyo/), AraGWAS (https://aragwas.1001genomes.org/) and GWApp (http://gwas.gmi.oeaw.ac.at) are available for Meta-GWAS analysis. Meta-GWAS have been reported in *A. thaliana* and soybean crop, but as per our updated knowledge, no Meta-GWAS has been reported in cotton crop. With the rapidly expanding volume of plant-GWAS data (even that of plant salt tolerance), meta-GWAS will be more popular for studying plants.

## Open questions

How feasible is mapping the causative genes/SNPs at exceptionally high resolution in order to grasp the utility of pleiotropy and how it can be applied in crop genetic improvement in the foreseeable future?

Can we predict crop responses to continuously changing ambient conditions by studying the genes and processes that contribute to phenotypic/physiological alterations?

How far are we from designing new climate-smart cultivars precisely by incorporating natural variants revealed by GWAS?

If a trait of interest is controlled by a rare variant, then why is that trait common in a large population but GWAS cannot find that associated rare variant?

## Concluding remarks and future perspectives

Cotton crop has evolved and adapted over millions of years, resulting in the accumulation of genetic based natural variations SNPs from various environments over time, resulting in many phenotypic functional variations. Recent advances in DNA sequencing have allowed for in-depth characterization of plant natural variations. These resources can be exploited with GWAS to link phenotypic variations to relevant genes or functional polymorphisms, providing insight into complex traits. Recent developments in the quantitative omics phenotypes (transcriptomics, metabolomics, and epigenomics) give rise to veritable alphabets of association studies TWAS, MWAS and EWAS collectively known as OWAS (omics wide association studies) coupled with high­throughput phenotyping platforms are beginning to yield unprecedented insights for the associated genes underlying agronomically important traits, expediting genomics assisted breeding. Consequently, we believe that developing more efficient GWAS computational algorithms would be highly desirable in this context. A better understanding of genetic variability at the SNPs level will help in *in-situ* conservation, characterization and utilization of diverse germplasm. GWAS will foster the breeding programs by expanding the accessibility to desired germplasm collections. Cotton GWAS’s ability to investigate the genetic architecture of complex traits has been established in several studies, and this number is projected to expand rapidly. However, most studies are of limited scope to genetic architecture’s main (additive) effect. This complexity is not only the result of differences in gene action, but also determined by ontogenic gene networks or even epigenetic effects, and the interaction with surroundings, and greatly changing environmental conditions. It will soon be possible to identify all underlying genes and their activities for major cotton traits, that will significantly speed up molecular breeding strategies. Although GWAS remains a valuable technique for fully using NGS technology breakthroughs, advances in statistical methods and genetic design can help to accomplish this goal. Diverse statistical methods can help us understand the genetic basis of complex features, but their differing assumptions remind us to careful selection of analysis methodology for each study or to combine allied methods. Improved population designs/core collections and new sequencing and statistical approaches may help identify and manipulate genetic elements generating quantitative variation. These novel designs will improve detection precision and accuracy by restructuring allelic spectra and diminishing confounding effects, which will help overcome intrinsic statistical hurdles.

## Author contributions

MY: Writing and idea development; HK: Proof reading and figures; QH: Language and proof reading; MR: Data compilation; MS: Data curation; YJ: Supervision; JR: Supervision. All authors contributed to the article and approved the submitted version.

## Funding

Zhejiang Provincial Natural Science Foundation of China (Grant No. Y21C130006) to YJ.

## Conflict of interest

The authors declare that the research was conducted in the absence of any commercial or financial relationships that could be construed as a potential conflict of interest.

## Publisher’s note

All claims expressed in this article are solely those of the authors and do not necessarily represent those of their affiliated organizations, or those of the publisher, the editors and the reviewers. Any product that may be evaluated in this article, or claim that may be made by its manufacturer, is not guaranteed or endorsed by the publisher.
